# CORTICAL CONTROL OF MOBILITY: MOVING TOWARD CLINICAL OUTCOMES AND INTERVENTIONS

**DOI:** 10.1093/geroni/igad104.0547

**Published:** 2023-12-21

**Authors:** Andrea Rosso, Qu Tian, Stephen Kritchevsky

## Abstract

A three-year series of pre-conference workshops at GSA a decade ago brought together leading scientists in the multidisciplinary field of brain, aging, and mobility. Since then, the field has advanced the understanding of motor control as a predominantly subcortical phenomenon to the attentional and cortical control of mobility and has extended the view of cognitive and motor functions as distinct to there being shared causes of cognitive and mobility declines. Emerging studies are further elucidating mechanisms of motor control (Holtzer, Baillargeon), demonstrating relations with important clinical outcomes, including dementia (Tian) and community mobility (Rosso), and exploring novel interventions on clinical and neurophysiological outcomes (Kahya), as demonstrated by the series of talks in this symposium. Roee Holtzer assesses prefrontal cortex efficiency over repeated trials of attention-demanding dual-task walking in older adults with and without multiple sclerosis. Emma Baillargeon explores the heterogeneity of prefrontal responses to walking among older adults and describes the relation of prefrontal response with performance metrics. Qu Tian demonstrates trajectories of gait variability measures over time predict future dementia risk. Andrea Rosso investigates prefrontal activation as a biomarker of gait automaticity and its relation to community mobility. Finally, Melike Kahya demonstrates brain stimulation through transcranial direct current stimulation as a potential intervention to improve standing balance during cognitively challenging tasks and examines the mechanisms of its action through dual-task cost and electroencephalograph (EEG) recordings. Together, these studies will provide an overview of the current understanding of cortical control of mobility and its implications for clinical outcomes and interventions.

